# ADMET Profiling of the Metallodrugs: A Comparative Review of Platinum, Palladium, Gold, Ruthenium, Copper, and Zinc Anticancer Complexes

**DOI:** 10.1155/bca/9578818

**Published:** 2026-08-12

**Authors:** Meshack Kotonto Tini, John Karanja, Lawrence Odiwuor Omwai, Daniel Onunga, Njogu M. Kimani, Charles O. Ochieng

**Affiliations:** ^1^ Department of Chemistry, Maseno University, Kisumu, Kenya, maseno.ac.ke; ^2^ Department of Physical Sciences, University of Embu, Embu, Kenya, embuni.ac.ke

**Keywords:** ADMET profiling, anticancer agents, essential metals, metallodrugs, noble metals

## Abstract

The successful clinical use of metal‐based anticancer agents depends heavily on a detailed understanding of their absorption, distribution, metabolism, excretion, and toxicology (ADMET) profiles. This review evaluates the ADMET properties of anticancer complexes involving platinum, palladium, gold, ruthenium, copper, and zinc. Unlike traditional organic medicines, the ADMET processes of these compounds are often complex and nonlinear, due to their unique coordination chemistry, ligand‐exchange kinetics, and dynamic speciation changes. The review highlights how structural differences in metal analogs influence their pharmacokinetics, systemic disposition, and distinct toxicity profiles. Ultimately, gaining a thorough understanding of these ADMET nuances is vital for designing next‐generation metallodrugs with optimized tissue distribution, minimal systemic toxicity, and improved therapeutic effectiveness.

## 1. Introduction

Metals in drugs introduce unique coordination geometries, variable oxidation states, and dynamic ligand substitution kinetics that cannot be imitated by organic compounds [1]. While organic small compounds have traditionally been the focus of traditional drug development pipelines, metallodrugs primarily serve as prodrugs that undergo structural changes in vivo, allowing for unique structure–activity interactions [2]. The clinical translation and therapeutic viability of these organometallic compounds rely heavily on their pharmacokinetic profiles, making the characterization of their absorption, distribution, metabolism, excretion, and toxicology (ADMET) pathways a prerequisite for understanding their complex systemic behavior [3]. Within this framework, absorption dictates the entry of the compound into systemic circulation based on its solubility and membrane permeability [4], which is immediately followed by distribution, the process governing the reversible movement of the metal complex between plasma and various tissues as influenced by blood flow and protein binding [5]. Once distributed, the complex undergoes metabolism involving chemical or redox alterations that yield active or inactive species [6], after which excretion defines the permanent elimination of the metal species from the body, occurring primarily via renal or biliary routes [7]. Overlaying this entire kinetic cycle is toxicology, which evaluates off‐target cellular damage, adverse drug reactions, and dose‐limiting liabilities throughout the body [8]. Given their propensity for ligand exchange and redox cycling, metallodrugs present highly complex, nonlinear ADMET profiles [9]. Changes in the metal center’s oxidation state directly alter its solubility, serum protein affinity, and clearance pathways, making early stage pharmacokinetic evaluation essential for modern bioinorganic drug designs [10].

## 2. ADMET Properties of Platinum (Pt) Complexes

Cancer chemotherapy has benefited significantly from platinum‐based drugs, beginning with cisplatin’s introduction [11]. However, challenges related to ADMET profiles, including toxicity and resistance, limit their clinical effectiveness [12]. Key platinum complexes (Figure S1) include cisplatin, carboplatin, oxaliplatin, nedaplatin, and miriplatin. Cisplatin, discovered in 1960, became the standard treatment for malignant tumors [13]. Carboplatin, a second‐generation agent, was developed with similar properties but offers reduced toxicity [14]. Oxaliplatin, a third‐generation drug, helps to overcome cross‐resistance while acting similarly to cisplatin [15]. The clinical utility of Pt(II) complexes principally cisplatin, carboplatin, and oxaliplatin is strictly bound to their systemic disposition and pharmacokinetic behavior [12], while lobaplatin and heptaplatin (Figure S2) are under clinical investigation for oral administration [16].

### 2.1. Absorption Profile of Platinum Complexes

Platinum compounds administered via intravenous injection [17] never suffer the solubility issues, and thus, the absorption stage of the ADMET profile is not applicable. Since the drug is introduced directly to systemic circulation, it achieves immediate 100% bioavailability; consequently, pharmacokinetics begins at the distribution phase immediately administered. Conversely, oral platinum (IV) prodrugs, such as satraplatin (Figure S3), rely on axial lipophilic ligands to cross the gastrointestinal mucosa via passive diffusion before undergoing systemic reduction [18].

### 2.2. Distribution of Profile Platinum Complexes

After systemic entry, platinum drugs quickly distribute into highly perfused organs such as the liver, kidney, and tumor tissues [10, 19]. Intracellular uptake is mediated via both active transport and passive diffusion. Cisplatin cellular accumulation depends on copper transporter 1 (CTR1) [18]. The significance of this transporter in drug accumulation is demonstrated by the correlation found between increased acquired resistance to cisplatin in ovarian cancer cell lines and decreased CTR1 expression [18]. On the other hand, oxaliplatin’s accumulation seems to be less reliant on CTR1, as its bulky diaminocyclohexane (DACH) moiety facilitates its transport through organic cation transporters (OCTs) [18].

The pharmacokinetic behaviors of three platinum analogs vary significantly, particularly with carboplatin displaying a higher distribution of platinum in ultrafiltrate plasma and a longer elimination time, which may contribute to its hematotoxicity [20]. At the subcellular level, comparative quantification reveals that intact oxaliplatin and its reactive metabolites accumulate predominantly within the mitochondria and cytosol, with a lower relative percentage localized within the nucleus [20]. Rapid transformation of oxaliplatin into platinum‐containing metabolites in cancer cells enhances its antitumor activity by disrupting mitochondrial functions. However, the persistence of these metabolites can lead to toxicity, reflecting a balance between efficacy and side effects [20].

Systemic transport is heavily modulated by protein‐binding ability [21]. Cisplatin and oxaliplatin bind extensively and irreversibly to plasma proteins, primarily serum albumin, transferrin, and gamma‐globulin within 24 h of administration [10]. This binding occurs preferentially at thiol (cysteine) and thioether (methionine) residues, effectively deactivating the metal center, reducing the concentration of free, therapeutically active drug and accelerating off‐target tissue retention [22]. Carboplatin exhibits lower, initially reversible protein binding, maintaining a higher plasma concentration of the ultrafilterable active drug over time [20].

### 2.3. Metabolism Profile of Platinum Complexes

#### 2.3.1. Aquation Kinetics of Platinum Complexes

Aquation is essential in the pharmacology of platinum drugs, involving the substitution of labile leaving groups with water to form reactive cationic complexes [18]. This activation increases the ability of platinum compounds to bind to DNA, driving their anticancer activity. The aquation rate influences cytotoxic efficacy and toxicity: Rapid aquation enhances activity but may elevate side effects, whereas slower aquation can reduce effectiveness [17]. High chloride levels in the extracellular environment suppress aquation, while lower intracellular chloride concentrations promote it [17, 18]. Among platinum drugs, cisplatin exhibits rapid aquation, carboplatin shows slower kinetics due to its stable chelated ligand, and oxaliplatin quickly forms active intermediates after cellular uptake [23].

#### 2.3.2. Adduct‐Mediated Cellular Uptake and Clearance of Platinum Complexes

The cellular absorption (influx) of carboplatin and oxaliplatin is heavily modulated by an intracellular chemical sink mechanism driven by DNA adduct formation. Upon entering the cytoplasm, these complexes covalently bind to the N7 position of purine bases, generating 1,2‐intrastrand cross‐links that effectively sequester the active platinum species within the nucleus [24]. This irreversible nuclear trapping shifts the intracellular equilibrium, maintaining a favorable concentration gradient that continuously promotes further cellular drug absorption over systemic detoxification routes [25].

Furthermore, the structural kinetics of these adducts directly influence net absorption velocity and plasma clearance; for instance, the bulky, repair‐resistant adducts of oxaliplatin limit intracellular saturation quickly, whereas the more easily repaired carboplatin adducts allow for sustained, long‐term cellular influx and prolonged plasma clearance profiles [5, 26].

#### 2.3.3. Prodrug Activation of Platinum Complexes

Platinum(IV) complexes function as kinetically inert prodrugs designed to resist premature extracellular aquation [27]. Intracellular activation requires chemical transformations involving a two‐electron reduction process, typically mediated by endogenous reducing agents such as ascorbic acid or reduced glutathione (GSH) [28]. This reduction cleaves the axial ligands, releasing the active, cytotoxic Pt(II) species directly within the intracellular environment [29]. Prodrugs thus can enhance drug stability in circulation, improve tumor accumulation, and reduce harm to healthy tissues by utilizing an intracellular activation mechanism, which focuses drug activity at tumor sites while decreasing reactivity in the bloodstream [30]. Future platinum drugs should focus on regulated activation mechanisms that remain inactive in systemic circulation but are highly responsive to specific biochemical signatures in the tumor microenvironment, including enzymatic overexpression and hypoxic conditions.

#### 2.3.4. Detoxification and Efflux of Platinum Complexes

Intracellular detoxification is driven by the conjugation of aquated platinum species to sulfur‐containing proteins. Platinum centers bind rapidly to GSH and metallothioneins (MTs), forming inert, water‐soluble mercapturic acid conjugates, which are less reactive [31]. These neutralized platinum–GSH conjugates are subsequently recognized and actively exported from the cell via ATP‐binding cassette (ABC) transporters, specifically multidrug resistance protein 1 (MRP1) [32]. For instance, Platinum–GSH conjugate efflux reduces intracellular accumulation, which aids in resistance and detoxification [18]. Intracellular sequestration by MTs and binding to cellular thiol pools can detoxify platinum compounds by blocking interaction with vital cellular targets [33]. This detoxification process is finalized when detoxified platinum conjugates are expelled from the body via biliary or renal pathways [18].

### 2.4. Excretion Pathways and Half‐Life of Platinum Complexes/Drugs

Cisplatin exhibits a biphasic elimination profile. The unbound, ultrafilterable platinum fraction is cleared rapidly via the kidneys, while the protein‐bound fraction displays a prolonged terminal half‐life extending over several days due to irreversible serum interactions [10]. Carboplatin demonstrates a longer intact drug half‐life (up to 12 h) because its cyclobutane dicarboxylate leaving group hydrolyzes at a significantly slower rate, resulting in a more predictable, cleared pharmacokinetic profile [10]. Nedaplatin, a cisplatin analog, shows reduced reactivity with blood proteins and is primarily eliminated via the kidneys, comprising nearly 50% of total platinum. Its pharmacokinetics are akin to carboplatin, with a biphasic elimination profile for ultrafilterable platinum [10]. Conversely, oxaliplatin possesses triphasic elimination kinetics characterized by a rapid initial distribution phase followed by a prolonged terminal elimination half‐life, driven by irreversible accumulation in erythrocytes [34]. The renal pathway is the primary route for the elimination of platinum complexes often eliminated as free metal ions or water‐soluble complexes [35]. Ultrafilterable, unbound platinum is cleared via glomerular filtration and active tubular secretion [35]. However, the accumulation of reactive, aquated intermediates within renal proximal tubule cells can impede rapid elimination, giving rise to long‐term tissue retention and prolonged physiological burdens [36]. Slow clearance of certain substances in patients can result in chronic toxicities, such as cumulative nephrotoxicity, and may pose environmental concerns for hospital and municipal wastewater systems [36]. There is a need for sustainable drug design principles that consider both the pharmacological and environmental impacts of metallodrugs, particularly regarding platinum excretion.

### 2.5. Toxicity Profiles of Platinum Complexes/Drugs

The dose‐limiting clinical toxicities of platinum compounds are tightly correlated with their unique pharmacokinetic profiles as indicated in Table S1. Cisplatin is primarily associated with severe, cumulative nephrotoxicity and ototoxicity due to its rapid aquation and preferential accumulation in renal tissues [37]. Carboplatin’s extended plasma half‐life and elevated ultrafiltrate concentration shift its primary toxicity profile toward bone marrow suppression and hematotoxicity [38]. Oxaliplatin’s rapid distribution and erythrocyte‐binding behavior trigger distinct, severe peripheral neurotoxicities [39].

## 3. ADMET Properties of Palladium (Pd) Complexes

Palladium complexes, sharing structural similarities with platinum, are being investigated as potential anticancer drugs due to the advantages in their ADMET profiles. They demonstrate faster kinetics and higher DNA reactivity compared to platinum drugs [40]. Notably, some palladium complexes are effective against cancers resistant to cisplatin and show reduced side effects, better cytotoxicity against various cancer cell lines, and improved aqueous solubility [41–43]. Additionally, they may offer enhanced interactions with DNA, leading to increased anticancer efficacy and reduced nephrotoxicity [41, 44, 45].

Palladium(II) complexes are emerging as promising alternatives to platinum‐based anticancer drugs due to their enhanced cytotoxicity and selectivity for cancer cells [46, 47]. These complexes exhibit strong stability and effective pharamcokinetics, as their robust Pd–C bonds ensure greater stability in biological environments, allowing them to effectively target cancer cells while minimizing harm to healthy tissues [46, 47]. Specifically, saccharinate‐containing compounds, coumarin–Pd(II) complexes (Figure S4), and those with primary 2‐pyridine thioamide ligands have shown significant anticancer potential [48]. While their rapid reactivity and capacity for metabolic disruption offer potent cytotoxic advantages, their clinical translation remains severely bottlenecked by poor systemically varied ADMET profiles.

### 3.1. Absorption and Distribution of Palladium (Pd) Complexes

The bioavailability of palladium (Pd) complexes is mostly dictated by their hydrophilicity–lipophilicity balance (log *p*). Favorable lipophilicity not only supports potential oral absorption [36] but is also crucial for overall antitumor activity [49]. Beyond simple diffusion, structural modifications allow certain Pd complexes to form self‐assembling nanostructures, which enhance tumor accumulation and extend circulation time > 12 h [50]. In vivo studies show that a dinuclear palladium (II)–spermine chelate (Pd_2_Spm) has better biodistribution than Pt (II) complexes in healthy mice, with lower tissue accumulation and increased selectivity for triple‐negative breast cancer cells [51]. Additionally, the bioavailability of palladium complexes is notably influenced by lipophilicity, which supports potential oral absorption due to favorable log *p* values [36]. Generally, certain palladium complexes may be suitable for oral administration due to their favorable lipophilicity profiles, offering an advantage over intravenous platinum therapies.

#### 3.1.1. Ligand‐Exchange Kinetics and Stability of Palladium (Pd) Complexes

Palladium(II) complexes present severe pharmacokinetic challenges, particularly concerning their absorption and distribution profiles occasioned by their excessive thermodynamic lability and ligand‐exchange kinetics. Palladium compounds have kinetics that are 102 to 105 times faster than analogous Pt complexes, which require specific chelating ligands to stabilize the palladium ion and minimize ligand‐exchange rates [52, 53]. In biological fluids, this hyper‐reactivity results in rapid, premature ligand exchange with common endogenous biomolecules [43]. Uncontrolled reactivity negatively affects drug absorption by degrading the drug matrix before it can pass through biological membranes, and it disrupts distribution through nonspecific binding to off‐target blood proteins, impeding therapeutic targeting.

To overcome barriers in drug distribution and absorption, it is essential to use strongly coordinating chelating ligands and stable leaving groups. These modifications stabilize the palladium ion, slowing hydrolysis and ligand‐exchange rates, thereby enhancing pharmacological effectiveness [46]. The selected chelating ligand significantly influences the complex’s lipophilicity and serum stability, impacting absorption and distribution. Although Pd(II) complexes are not widely used clinically, they serve as valuable models to understand metallodrug kinetics and inform the design of stable next‐generation metallodrugs with favorable ADMET profiles.

### 3.2. Metabolism of Palladium (Pd) Complexes

Palladium(II) complexes have been studied for their metabolic effects, including cell death, antioxidant defense, and alterations in metabolic pathways. They induce apoptosis through mechanisms like caspase‐3 activation, increase reactive oxygen species (ROS), and decrease GSH levels, leading to oxidative stress and endoplasmic reticulum (ER) stress [54, 55]. Alterations in various metabolites, such as choline and amino acids, have also been observed [54]. Polynuclear palladium complexes can interact with multiple biological targets, potentially reducing side effects [54]. Their catalytic properties may aid in biochemical reactions, offering potential for anticancer therapy due to their significant interference with cellular metabolism and induction of apoptosis in cancer cells [56, 57]. However, the exact mechanisms by which Pd(II) complexes alter metabolism are not fully understood, highlighting a significant research gap in this area.

### 3.3. Excretion Pathways and Half‐Life of Palladium (Pd) Complexes

A significant limitation in evaluating Pd(II) complexes is the lack of comprehensive pharmacokinetic data, especially regarding excretion pathways and half‐life kinetics. Unlike well‐understood platinum‐based drugs, empirical data on hepatobiliary and renal clearance remain limited [35]. The kinetic profiles show contradictions observed in vivo, particularly with a dinuclear palladium(II)–spermine chelate (Pd_2_Spm) that exhibited biphasic serum kinetics, revealing a terminal half‐life of 20.7 h, while unbound palladium showed an even longer half‐life of 35.5 h [51]. Typically, free metallodrugs are cleared more rapidly than their bound forms, suggesting either rapid re‐equilibration from tissues or possible renal filtration failures. These prolonged half‐lives indicate significant systemic persistence of palladium species. Despite detailed in vitro toxicity and tissue distribution studies, the metabolic clearance and elimination pathways remain poorly understood [40], creating a critical knowledge gap that complicates predictions of tissue accumulation and chronic toxicity. Continued research into excretory mechanisms is essential for establishing safety margins and informing clinical dosing strategies.

### 3.4. Toxicity Profiles of Palladium Complexes

Palladium‐based compounds are roughly ten times less toxic than platinum‐based compounds, according to toxicological studies conducted on rats [46]. However, due to rapid ligand‐exchange kinetics and insufficient stability of some Pd (II) complexes in biological fluids, certain challenges still exist that impede successful in vivo efficacy and proper management of potential toxicity [46].

The Pd–saccharinate complex selectively exhibits antiproliferative properties against UACC‐62, without causing mutations or affecting nontumorigenic keratinocyte growth (HaCaT) [46, 58]. The selective activity results can be corroborated by its nonmutagenic potential in the Ames test and lack of effect on HaCaT cell growth [58]. Similar to cisplatin, certain palladium complexes, like Pd–Py–OEt and Pd–Py–NEt_2_ (Figure S4), have cytotoxicity but are noticeably more selective for cancer cell lines than normal cell lines [59]. These compounds showed superior potency and selectivity compared to cisplatin, with high nontumor cell selectivity exhibited as cytostatic effects, with apoptosis as the primary cell death mechanism [59].

The ADMET profile of palladium complexes is thus influenced by tightly bound chelate ligands and careful ligand selection, leading to decreased toxicity and enhanced selectivity [46]. The relationship between the chemical structure and biological results underscores the significance of sensible ligand design in obtaining palladium complexes with drug‐like ADMET properties.

## 4. ADMET Properties of Gold (Au) Complexes

In the search for novel cancer therapeutics, gold complexes have emerged as a highly promising class of metallodrugs, offering distinct advantages over conventional platinum‐based agents with more favorable ADMET profiles [60]. Gold complexes have unique methods of action by targeting vital biological components such as mitochondria and the enzyme thioredoxin reductase (TrxR), in contrast to platinum medications that mainly target DNA. ROS are produced as a result of this targeted inhibition, which upsets cellular redox balance and ultimately causes apoptotic cell death [60, 61].

Gold(III) complexes have become increasingly popular as potential alternatives to conventional platinum‐based drugs due to their isostructural and isoelectronic properties to Pt(II). However, their poor stability under physiological settings is a significant obstacle to their clinical development [62]. Such limitations raise a critical question regarding the optimal structural requirements necessary to ensure both robust stability and favorable ADMET properties. Recent structural innovations have sought to address this challenge where conformationally restricted Au(III) complexes and binuclear biphenyl organogold complexes demonstrated potent anticancer activity [48]. Additionally, novel Au(I) complexes featuring alkylated phosphaadamantane ligands have shown great promise as thermoresponsive anticancer agents, specifically targeting human colon cancer cells [48].

### 4.1. Absorption Profile of Gold (Au) Complexes

#### 4.1.1. The Role of Protein Binding in Systemic Absorption and Transport

The systemic absorption and retention of gold organocompounds in the bloodstream are primarily influenced by their interaction with plasma proteins. Their biological activity and cellular uptake are linked to selective binding with free cysteine residues, which can disrupt protein function, inhibit enzymes, and lead to targeted cell death [63, 64]. Analyzing structural features of known gold/protein adducts aids in understanding these mechanisms.

Studies show that most absorbed, protein‐bound gold attaches to albumin, with minimal traces in blood cells [65]. Gold complexes and nanoparticles (AuNPs) (Figure S5) interact with bovine serum albumin (BSA) through hydrophobic interactions, hydrogen bonding, and electrostatic forces, which depend on the characteristic of the gold complex’s surface [62, 66]. For instance, electrostatic forces influence citrate‐coated surfaces, while steric mechanisms help stabilize BSA [66]. Interactions affecting BSA’s secondary structure decrease α‐helix content while increasing β‐turns and disordered structures. This mild, reversible binding affinity allows BSA to function as an effective endogenous carrier, enhancing systemic drug distribution and preventing premature inactivation before reaching tumor sites [66].

The oxidation state of gold shifts during absorption, with Au(III) molecules often degrading to Au(III) and Au(I) species [63]. In chrysotherapy patients (gold salt treatment), Au(I) species circulate in serum, tightly bound to albumin and globulins [65]. The stability of these Au(I)–protein complexes arises from gold’s strong affinity for sulfur‐containing amino acids in biological macromolecules [67].

#### 4.1.2. Physiological Stability as a Prerequisite for Efficient Absorption

The absorption efficiency of Au(III) complexes is limited by their instability in physiological conditions, often being reduced to inactive forms or metallic gold, which lowers bioavailability [62]. Ensuring the structural integrity of these complexes for sufficient absorption and targeting is a key challenge in developing gold‐based drugs. Advanced coordination chemistry has significantly improved the physiological stability of gold (III) complexes through the use of cyclometallation and multidentate ligands [62]. Cyclometallation enhances compound stability by creating a strong covalent *σ*(M–C) bond, which enhances thermodynamic stability, while multidentate ligands form stable chelate rings, preventing premature dissociation in biological environments [68].

Successful synthesis of Au(III) dithiocarbamate complexes (Figure S6) illustrates an effective strategy for enhancing stability, demonstrated by their resilience in phosphate‐buffered saline and biological reductant‐rich environments [69]. Through rational ligand design, controlling the metal center’s redox state and reactivity can be used to protect the gold complex from premature degradation. This approach serves as a blueprint for overcoming stability challenges, ensuring that the metallodrug remains intact for optimal absorption and therapeutic distribution.

### 4.2. Distribution Profile of Gold (Au) Complexes

Achieving an optimal biodistribution profile is crucial in developing gold‐based therapeutics, focusing on prolonged systemic circulation, efficient blood transport, and selective accumulation in target tissues. Recent advancements in coordination chemistry and nanotechnology have improved these complexes’ ability to overcome biological barriers and target specific disease sites. The distribution of small‐molecule gold therapeutics is significantly influenced by their physicochemical properties, affecting their blood transport and tissue partitioning [62]. Gold(III) dithiocarbamate complexes (Figure S6) exhibit favorable blood transport and biodistribution, driven by an optimal balance between hydrophilicity and lipophilicity. This amphiphilic balance maintains stability and ensures membrane permeability with partition coefficients (log *p*) from −1 to 3, facilitating both intestinal absorption and extensive tissue distribution [69]. As a result, these complexes possess an excellent pharmacokinetic profile, leading to effective anticancer and antibacterial actions in vitro and in vivo [62].

To address the limitations of traditional small‐molecule drugs, NPs formulations, particularly AuNPs, are employed due to their nanoscale design (10 to 100 nm) [70]. These NPs enhance drug biodistribution by extending circulation time and reducing off‐target toxicity [71]. Surface modifications, such as PEGylation, improve circulation duration but lead to significant long‐term accumulation in organs like the liver and spleen, raising concerns about potential late‐stage toxicities and requiring further study [72, 73]. Long‐term retention in the mononuclear phagocyte system (MPS) may result in localized inflammation and liver function disruption.

Active targeting using AuNP enhances site‐specific tumor localization by conjugating them with targeting moieties, such as cetuximab, which targets the epidermal growth factor receptor (EGFR) [74]. This method increases local drug efficacy by concentrating the gold payload in tumor cells, reduces off‐target toxicity by lowering gold concentration in healthy tissues, and is effective against drug resistance by bypassing cellular efflux mechanisms [75]. Overall, optimizing the biodistribution of gold‐based therapeutic agents, including AuNPs, is crucial for maximizing their clinical effectiveness.

### 4.3. Metabolism Profile of Gold (Au) Complexes

Gold complexes exhibit a complex metabolic profile involving biotransformations, oxidation‐state changes, and ligand‐exchange reactions, which influence their activation and therapeutic effectiveness. Unlike platinum‐based agents that directly target DNA, gold complexes act as metabolic modifiers and prodrugs, focusing on key cellular enzyme systems [3, 76].

#### 4.3.1. Prodrug Biotransformation and Ligand Exchange

A key metabolic characteristic of gold complexes is their activation dependence on specific biological conditions, influenced by coordination geometry, ligand types, and metal oxidation states [76]. Different oxidation states lead to distinct metabolic pathways; for instance, Au(I) drug auranofin (Figure S6) acts as a prodrug that is metabolically converted to active species through ligand exchange [76]. In contrast, gold(III)–dithiocarbamate complexes are metabolically reduced to Au(I) intermediates through redox transformations, allowing them to interact with oxidized proteins [77].

#### 4.3.2. Enzyme and Organelle Targeting Driven by Metabolism

Active gold species, upon metabolic processing, bind strongly to sulfur‐ and selenium‐containing biomolecules, disrupting cellular redox balance and organelle integrity [78]. A key effect is the inhibition of TrxR by gold(I) complexes like auranofin, which binds to selenocysteine in TrxR, leading to oxidative stress and apoptotic cell death [79–81]. This mechanism is also seen in dual‐gold(I) complex QB1561 (Figure S6), inhibiting TrxR and mitochondrial respiration [79]. Additionally, digold phosphine complexes accumulate in mitochondria, causing oxidative phosphorylation disruption and generating DNA–protein cross‐links [78, 82]. In contrast, gold(III)–dithiocarbamate complexes inhibit proteasomal activity, leading to acute stress and tumor growth suppression [3, 83].

Gold complexes exhibit a dual metabolic nature influenced by their microenvironment and chemical structure. While they generally induce cytotoxic ROS production in tumor cells, some gold(III)–dithiocarbamate complexes can display antioxidant properties, promoting a balanced redox state by lowering oxidized protein levels [69, 84]. This metabolic flexibility indicates potential therapeutic uses in addressing noncancerous oxidative stress conditions [69]. Generally, gold complexes leverage alternative metabolic pathways, particularly focusing on TrxR, the proteasome, and mitochondrial machinery, to circumvent drug resistance seen with platinum agents and DNA‐targeting therapies [76].

### 4.4. Excretion Pathways of Gold (Au) Complexes

The excretion profile of gold complexes represents a significant pharmacological advantage, particularly when contrasted with traditional platinum‐based therapies. While platinum drugs are primarily eliminated via renal clearance often resulting in severe, dose‐limiting nephrotoxicity, gold complexes preferentially utilize hepatobiliary processing, leading to rapid fecal elimination [69, 85]. This excretion pathway is heavily influenced by the physicochemical properties of the metallodrugs, including molecular size, coordination geometry, and surface modifications [86].

Preclinical research in animal models demonstrates that the rapid fecal elimination of gold(III)–dithiocarbamate complexes significantly reduces organ‐specific and systemic toxicities [69, 85]. These complexes avoid kidney involvement, reducing damage to normal tissues, maintaining organ function, and exhibiting low acute toxicity in in vivo xenograft models [87]. This efficient biliary‐fecal clearance route is similarly advantageous for gold‐based NPs and nanoclusters, ensuring timely elimination from the body and mitigating long‐term chronic accumulation concerns [88, 89]. Establishing a link between the excretion pathway and reduced tissue toxicity suggests a design strategy for next‐generation metallodrugs. By favoring fecal excretion, these drugs can potentially avoid the severe renal complications linked to heavy‐metal chemotherapies.

### 4.5. Toxicity Profile of Gold (Au) Complexes

Gold complexes and nanotechnology have therapeutic potential, but their clinical application depends on resolving important ADMET issues. These substances exhibit potent antiproliferative activity and tumor cell selectivity although their systemic safety profiles remain complex [76, 90–92]. The therapeutic selectivity of these molecules is linked to mechanisms that alter cellular survival, such as a high affinity for sulfur‐containing biomolecules, inhibition of TrxR, disruption of redox homeostasis, and induction of oxidative stress and apoptosis [76]. Optimizing these toxicodynamic interactions is crucial for enhancing selectivity, improving pharmacokinetics, reducing off‐target damage, and mitigating systemic toxicity [64].

Gold‐based therapeutics face significant hurdles due to their late‐stage toxicological profiles, organ accumulation, and systemic risks. While some gold complexes exhibit mild toxicity, others, like digold phosphine complexes, show severe cardiotoxicity, impeding clinical development [93]. Traditional auranofin therapy remains restricted by toxicity issues [94]. Nanomedicines such as PEG–AuNPs have beneficial distribution kinetics, yet suffer from poor excretion, leading to harmful long‐term accumulation in organs and associated cytotoxicity [72, 73, 95]. Some derivatives like gold(III)–dithiocarbamate, despite showing promising short‐term profiles with minimal organ toxicity, may conceal long‐term risks [72, 73, 95]. Long‐term safety studies are essential for the clinical translation of nanotechnologies due to potential delayed toxic effects. Current strategies aim to enhance the stability of gold species, especially volatile Au(III) complexes. Techniques to reduce premature degradation and off‐target reactivity include cyclometallation to strengthen coordination and the use of multidentate chelating ligands to prevent systemic gold shedding.

Gold complexes represent a highly promising and strategically advantageous class of metallodrugs for oncology, primarily due to their favorable and tunable ADMET profiles, which offer distinct advantages over conventional platinum‐based agents like cisplatin. The sophisticated coordination chemistry of gold allows for the engineering of drugs with high stability, optimal bioavailability, highly selective anticancer mechanisms, desirable excretion profile, and a low‐toxicity clearance pathway. The gold complex platform offers a versatile strategy for developing next‐generation metallodrugs to combat cancer, especially drug‐resistant malignancies, despite long‐term toxicity concerns in some nanoformulations and complexes.

## 5. ADMET Properties of Ruthenium (Ru) Complexes

Ruthenium complexes are promising metal‐based therapeutics known for their antimetastatic properties and ability to combat platinum resistance [96]. However, fully realizing their clinical potential necessitates a thorough assessment of their ADMET profiles. These compounds are theorized to preferentially accumulate in cancerous tissues, facilitating tumor‐specific activation [96, 97]. This selective activation leverages the acidic, reducing microenvironment of tumors to transform the inactive Ru(III) prodrug into the active, cytotoxic Ru(II) species, thus reducing systemic toxicity [97]. However, optimizing the ADMET properties of ruthenium complexes presents significant pharmacokinetic challenges, particularly with delivery and targeting, which can lead to severe off‐target toxicities if tumor‐specific activation is not accurately designed [98].

The relationship between structural design and toxicological effects is exemplified by how ruthenium frameworks induce cell death through various mechanisms. Photoactivatable ruthenium complexes aim to generate localized ROS and target specific proteins upon light exposure, minimizing systemic damage [48]. Clinical trials are assessing candidates such as NAMI‐A, KP1019, KP1339, and TLD1433 (Figure S7), focusing on maximal tolerated doses and pharmacokinetic profiles [99]. Preclinical complexes, such as Ru‐Cyn‐1 and [Ru (dip)_2_(PPβC)]PF_6_ (Figure S8), illustrate distinct toxicity pathways, with Ru‐Cyn‐1 accumulating in mitochondria to produce Type I and II ROS and [Ru (dip)_2_(PPβC)]PF_6_ (Figure S8) inducing apoptosis via mitochondrial DNA topoisomerase I inhibition [100]. Addressing the ADMET challenges of ruthenium therapies requires improved rational drug design for better tumor selectivity and innovative drug delivery techniques.

### 5.1. Absorption Profile of Ruthenium (Ru) Complexes

The absorption and systemic biodistribution of ruthenium (Ru) complexes mark a significant shift from traditional platinum‐based chemotherapy, offering notable pharmacokinetic benefits for tumor targeting [97, 101]. Unlike platinum drugs, which face rapid clearance and off‐target toxicity, Ru(III) complexes demonstrate an advantageous pharmacokinetic profile with a small volume of distribution, low clearance, and extended elimination half‐life [97]. This allows for sustained therapeutic presence in the bloodstream [102]. Their absorption and transport are greatly influenced by high affinity for plasma proteins, such as serum albumin and transferrin [103], with clinical candidates NAMI‐A and KP1019 utilizing this capacity for effective delivery to tumors [103, 104].

### 5.2. Distribution Profile of Ruthenium (Ru) Complexes

Ruthenium complexes show selective biodistribution and uptake in malignant cells by mimicking iron. Tumor cells overexpress transferrin receptors (TfR1), which enables ruthenium compounds to enter tumors through receptor‐mediated endocytosis by binding to transferrin [17, 96, 105]. This results in greater accumulation in cancerous tissues while preserving healthy cells, which have lower TfR1 expression [105]. However, subtle structural variations among ruthenium frameworks may significantly affect their biodistribution and pharmacological profiles. For instance, KP1019 is rapidly internalized, leading to high intracellular concentrations that disrupt DNA and mitochondria, triggering apoptosis and autophagy [97, 103, 106]. In contrast, NAMI‐A shows poor intracellular penetration and primarily localizes extracellularly, binding to integrins to inhibit angiogenesis and tumor metastasis without inducing direct cell death [97, 103]. Such findings highlight how modifications to the ruthenium coordination sphere can fine‐tune a drug’s ADMET properties for targeted therapeutic outcomes.

### 5.3. Metabolism of Ruthenium (Ru) Complexes

The metabolic profile of ruthenium (Ru) complexes highlights a biotransformation strategy that focuses on tumor‐specific activation and designed ligand‐exchange kinetics. Unlike conventional therapies relying on hepatic enzymatic degradation, the metabolism of these complexes is influenced by the tumor microenvironment and coordination chemistry, regulating their conversion from inert transport vehicles to reactive, cytotoxic agents [105, 107–109].

#### 5.3.1. Reductive Metabolism and Prodrug Activation

The design of ruthenium drugs hinges on a prodrug activation by reduction mechanism. Initially, ruthenium is delivered as the stable Ru(III) form to avoid early reactivity and damage [110–112]. In the tumor microenvironment, characterized by hypoxia and acidity, Ru(III) is reduced to the reactive Ru(II) species [113, 114], facilitated by intracellular reducing agents like GSH and low pH typical of cancer cells [115]. Additionally, some candidates like TLD1433 (Figure S10) may utilize light‐induced metabolism for activation, producing reactive singlet oxygen and ROS directly at tumor sites [61]. In vivo findings suggest that ruthenium metabolism is more complicated than in vitro models, notwithstanding the tumor‐selective metabolic model’s potential. Clinical research on trans‐[tetrachlorobis(1H‐indazole)ruthenate(III)] {BOLD‐100 (KP1339)} (Figure S7) shows a discrepancy that despite its strong in vivo efficacy, a significant amount of the drug remains in the unreduced Ru(III) state [116]. This emphasizes the need for a thorough validation of metabolic activation pathways in living systems and the critical role that the in vivo tumor microenvironment plays in affecting medication metabolism.

#### 5.3.2. Ligand Exchange and Coordinate Metallocatabolism

Beyond electronic redox reduction, the metabolism of Ru(III) and Ru(II) complexes involves ligand‐exchange kinetics, enabling the metal to shed its ligands and hydrolyze into a biologically active form [105, 117, 118]. By manipulating ligand types and charges, developers can optimize aquation kinetics similar to platinum‐based drugs [116, 119]. The metabolic hydration rate determines when and where the ruthenium center is available to cross‐link with biological macromolecules [105, 117, 118]. Once ligand exchange activates the coordination sphere, metabolized ruthenium species can engage critical intracellular targets. This enables binding to nuclear DNA and G‐quadruplex structures, inhibiting telomerase and disrupting cancer cell replication [48]. Additionally, structural changes lead to metabolic collapse through ER stress, mitochondrial membrane disruption, and ROS generation, promoting apoptotic cell death [105, 107–109].

### 5.4. Excretion Pathways of Ruthenium (Ru) Complexes

The elimination phase of ruthenium (Ru) complexes, particularly their excretion and clearance kinetics, is crucial in ADMET profiling. Unlike many compounds, these heavy‐metal complexes are not metabolized into volatile substances; rather, their clearance relies on renal filtration or biliary excretion. Variations in their coordination and peripheral ligand structures influence these pathways, affecting systemic clearance and the potential for tissue retention and toxicity [35].

#### 5.4.1. Renal Clearance Kinetics and Elimination Divergence

The divergent elimination half‐lives (*t*_½_) and clearance rates of KP1019 and NAMI‐A highlight the critical role of structural design in excretion kinetics. KP1019 has a low systemic clearance rate and a prolonged half‐life, leading to significant tissue retention and potential cumulative toxicity risks [120]. In contrast, NAMI‐A exhibits rapid renal clearance and swift excretion, reducing chronic toxicity risks but also limiting its therapeutic window [117]. Thus, the structural differences result in a pharmacokinetic trade‐off of slow clearance for KP1019 versus rapid elimination for NAMI‐A.

#### 5.4.2. Biodistribution Decay and Organ Accumulation

Understanding drug excretion involves examining how drug concentrations decline in different body compartments. In studies with Balb/c mice bearing CT26 tumors, BOLD‐100 (NKP‐1339) showed initial widespread distribution, notably in serum, liver, brain, bones, and muscles [121]. As excretion progressed, most organ concentrations decreased, but the kidneys retained high ruthenium levels, indicating that they are the primary excretion route [35, 121]. Furthermore, tumor tissues maintained higher metal concentrations compared to healthy organs, suggesting effective targeting with favorable elimination kinetics [121]. The differences in the retention of KP1019, rapid clearance of NAMI‐A, and renal persistence of BOLD‐100 highlight the nonuniformity of excretion within the ruthenium metallodrugs. To ensure long‐term safety predictions and avoid late‐stage organ toxicities, thorough mass balance profiles and detailed fecal versus urinary excretion studies are essential for the clinical application of new ruthenium‐based compounds.

### 5.5. Toxicity Profile of Ruthenium (Ru) Complexes

The toxicological profile of ruthenium (Ru) complexes marks a significant advancement in metal‐based oncology, offering a wider therapeutic index compared to traditional platinum‐based agents. Ruthenium complexes exhibit reduced systemic toxicity and nonspecific cytotoxicity under normal physiological conditions, making them promising candidates to alleviate severe chemotherapy side effects [105, 117]. This advantageous safety profile is largely due to their toxicodynamic selectivity, as ruthenium compounds preferentially accumulate in neoplastic tissues by exploiting overexpressed transferrin receptors on malignant cells, thereby protecting healthy cells from off‐target effects [117].

Despite their safety advantages, certain ruthenium complexes face toxicological issues due to their stability and formulation. While Ru(III) complexes are tolerated, they often exhibit poor solubility and physiological stability, risking off‐target toxicity if they degrade before reaching tumors [117]. Conversely, organometallic frameworks like RAPTA complexes (Figure S9) are designed for very low systemic toxicity, reducing side effects compared to conventional treatments [120]. The toxicity profile of certain ruthenium compounds, particularly NAMI‐A, is distinct from traditional platinum drugs. NAMI‐A specifically targets tumor cell subpopulations involved in metastasis while exhibiting low toxicity toward the primary tumor. This approach redefines metallodrug design goals, favoring nontoxic anti‐invasive actions over systemic cytotoxicity [103, 117]. As a result, ruthenium complexes are well suited for combination therapies, helping to reduce metastasis without increasing the systemic toxicity from other cytotoxic agents.

## 6. ADMET Properties of Copper (Cu) Complexes

Copper is a crucial trace element in cellular redox balance that has gained traction as a biometal for novel anticancer therapies. Copper(II)‐based complexes are effective for inducing DNA damage, cell cycle arrest, and apoptosis [122–124]. However, successful clinical application requires careful optimization of their ADMET properties, influenced by their coordination chemistry, ligand‐exchange kinetics, and physicochemical traits, which also determine their pharmacokinetics and safety in biological systems [125]. A key mechanism of action involves their participation in redox cycling, which leads to the formation of ROS, crucial molecules for initiating cell apoptosis and overall cytotoxic effects [122–126].

Promising candidates under evaluation include casiopeínas, elesclomol, copper‐disulfiram (Cu‐DSF) (Figure S10) and specific copper compounds that inhibit topoisomerase [127]. Casiopeínas, particularly Casiopeinas III‐ia, demonstrate a strong activity against breast, lung, and colon cancers by inducing mitochondrial dysfunction and generating ROS [124, 128, 129]. Their bioavailability is influenced by lipophilicity and structural stability, allowing them to cross biological membranes. Once in circulation, maintaining complex integrity is crucial for targeting malignant tissues [122, 123]. Following cellular uptake, Cu^2+^ often reduces to Cu^+^, generating ROS that can induce apoptosis and mitochondrial dysfunction [125, 126]. Structural designs aim to balance targeted cytotoxicity and systemic toxicity. For example, the Casiopeínas use mixed ligand sets for improved uptake [128, 129], while candidates like elesclomol and Cu‐DSF (Figure S10) leverage ionophore strategies to guide copper distribution and alter toxicity [127]. Fine‐tuning these factors is essential for minimizing off‐target damage and achieving clinical viability [124].

### 6.1. Absorption Profile of Copper Complexes

The bioavailability and uptake of copper complexes are influenced by their lipophilicity, coordination geometry, and stability. Lipophilic complexes, such as casiopeínas and Cu‐DSF (Figure S10), enhance cellular internalization through passive diffusion and active transport, allowing them to circumvent cellular copper regulatory mechanisms [124, 130–132]. This gives them an advantage over hydrophilic complexes, which depend on the human copper transporter 1 (hCTR1) often down‐regulated by high intracellular copper levels [133–135]. Hydrophilic formulations can be absorbed through endocytic pathways associated with cancer cell interactions [136]. Additionally, exogenous chelators can significantly lower copper bioavailability, affecting absorption and therapeutic efficacy [130].

### 6.2. Distribution Profile of Copper Complexes

Once in systemic circulation, copper compounds interact with biomolecules, maintaining coordination integrity to prevent ligand dissociation before reaching target tissues [122, 123]. After crossing the plasma membrane, their lipophilicity aids intracellular distribution [124], with many functioning as ionophores to transport copper across organelle membranes, inducing targeted toxicity [131]. Elesclomol exemplifies this by selectively delivering copper to mitochondria, leading to oxidative stress and cell death in mitochondrially dependent tumors [137]. Moreover, subcellular distribution is crucial for the function of copper complexes as prodrugs. Once localized in specific compartments, they are converted to toxic Cu(I) by proteins like ferredoxin 1 (FDX1). This accumulation enables them to bind and inhibit essential biomolecules, produce toxic ROS, or disrupt survival pathways, such as interfering with the HIF complex through the CCS chaperone [137, 138]. This distribution allows targeting DNA in the nucleus or inducing oxidative stress in mitochondria, leading to a more effective intracellular delivery compared to traditional metal‐based drugs like platinum complexes [131].

### 6.3. Metabolism of Copper Complexes

#### 6.3.1. Copper(II) Homeostasis and Prodrug Activation in Cancer

The cytotoxicity of copper complexes in cancer treatment is fundamentally linked to their ability to exploit the cell’s natural redox chemistry and disrupt native copper homeostatic pathways. The body tightly regulates copper levels, primarily in the liver, where the transporters ATP7A and ATP7B manage intracellular concentrations and biliary excretion, while MT acts as a storage protein [125, 139]. Aggressive tumors often exhibit low‐oxygen conditions, requiring copper complexes to disrupt this balance, releasing active Cu (I) or ROS, concentrating cytotoxic effects in malignant cells [122, 126, 139]. For example, thiosemicarbazone copper complexes exploits hypoxia for its accumulation, causing oxidative stress, DNA damage, and cell death in low‐oxygen cells, while acting as an antioxidant in nonhypoxic cells [140].

#### 6.3.2. Oxidative Mechanisms and Targeted Copper Delivery

The primary action of copper complexes against cancer involves generating oxidative stress, which causes DNA, protein, and lipid damage, leading to apoptosis [125, 139]. Specific agents like elesclomol, a copper ionophore, and certain oxindolimine–Cu (II) enhance this by delivering copper directly to mitochondria, resulting in copper overload and triggering unique cell death pathways like ferroptosis [139]. This strategy is particularly effective in tumors with high mitochondrial metabolism, such as melanoma [132, 139]. Additionally, copper NPs are noted for their oxidative mechanisms that induce cytotoxicity and apoptosis in cancer cells, including breast and colon cancers [141].

#### 6.3.3. Nonoxidative Strategies and Molecular Targets of DNA of Copper (Cu) Complexes

Intracellular metabolism of copper complexes (e.g., with DSF/copper and Caspiopeínas) employs nonoxidative strategies, such as ligand exchange, to form active species that target critical molecular sites [142, 143]. These activated species act as topoisomerase inhibitors [144, 145], causing irreversible DNA damage and triggering cell death pathways [124, 142, 143]. Notable examples include copper (II) complexes with ligands such plumbagin, phenanthroline, and thiosemicarbazone that disrupt the CCS chaperone pathway, in turn inhibiting HIF‐1 and consequently blocking protumor signaling and VEGF expression [146]. This highlights the therapeutic potential of copper complexes in cancer treatment through multiple mechanisms affecting uptake, trafficking, and key proteins.

### 6.4. Excretion Pathway of Copper Complexes

The clearance and excretion of copper complexes are essential for their systemic half‐life and organ toxicity prevention. Copper, as an essential biometal, relies on endogenous pathways like hepatic clearance (fecal) and biliary excretion for its removal [147, 148]. However, synthetic coordination complexes may disrupt these natural processes. The excretion rate and route of copper complexes depend on their stability and hydrophilicity. Stable, hydrophilic complexes or their metabolites are filtered through the glomerulus for renal excretion [148, 149], while lipophilic complexes may release copper ions that bind to proteins, leading to slower hepatic‐biliary clearance [148, 150]. Balancing these kinetics is crucial to ensure targeted tumor accumulation while preventing toxic buildup in the liver and kidneys.

### 6.5. Toxicity Profile of Copper Complexes

#### 6.5.1. Mechanisms of Cytotoxicity and Off‐Target Risks

The primary mechanism of copper‐induced toxicity involves intracellular redox cycling, leading to the generation of ROS. This oxidative stress can damage cellular components, causing DNA breaks, protein denaturation, and lipid peroxidation [151]. Clinical candidates like elesclomol demonstrate the potential for targeted mitochondrial collapse in tumor cells but risk oxidative damage in healthy tissues if their biodistribution is not properly managed [131, 132].

#### 6.5.2. Endogenous Defense and Homeostatic Regulation

To mitigate the risks of copper toxicity, the human body utilizes a sophisticated detoxification system that copper concentrations well below toxic thresholds. This includes storing excess intracellular copper through MTs [152] and employing P‐type ATPases (ATP7A and ATP7B) to pump out surplus copper from cells [153]. Additionally, free radicals generated by copper redox reactions are neutralized by antioxidants such as copper, zinc–superoxide dismutase (Cu, Zn–SOD) [153].

#### 6.5.3. Systemic and Organ‐Specific Toxicity Endpoints

When synthetic copper complexes overwhelm endogenous defenses, they cause systemic and organ‐specific toxicities, particularly hepatotoxicity and nephrotoxicity, due to the liver and kidneys’ roles in copper regulation [154, 155]. This leads to functional impairment or tissue necrosis [154, 155]. Additionally, chronic ROS‐mediated DNA damage raises concerns about severe genotoxicity and secondary malignancies [67]. Unstable complexes in circulation also pose a risk of hematotoxicity, manifesting as anemia or leukopenia [156].

To overcome these challenges, recent chemical redesigning approaches aim to address systemic limitations in drug toxicity by exploring noncanonical death pathways. A prominent example is HydroCuP, a phosphino copper(I) complex that avoids traditional apoptotic toxicities by selectively inducing ER stress and paraptosis [157]. This strategy permits the compound to maintain a favorable safety profile with minimal systemic toxicity. To effectively reduce the toxicity of copper‐based drugs, a thorough understanding of copper homeostasis is essential for designing stable, selective coordination spheres that activate within the tumor microenvironment.

In summary, copper complexes can combat platinum drug resistance by targeting molecular pathways, reducing drug accumulation, and enhancing DNA repair, unlike platinum drugs that primarily form covalent DNA adducts. Copper’s redox capacity allows it to catalyze oxidative reactions, producing ROS, which can damage and apoptosis cancer cells, offering a potential therapeutic solution; elevated copper levels can selectively target tumor cells requiring high copper metabolism, potentially reducing toxicity to normal cells, as they are often linked to tumor growth and angiogenesis. Copper complexes target multiple molecular targets, offering a versatile cancer therapy approach. Research and clinical validation explore fewer off‐target effects and toxicity compared to platinum drugs.

## 7. ADMET Properties of Zinc (Zn) Complexes

Zinc complexes show potential as anticancer agents, influenced by ligand choice and structural characteristics. The complexes are designed for enhanced bioavailability, reduced toxicity, and increased tumor selectivity compared to conventional chemotherapy like cisplatin [158]. While no zinc‐based anticancer drugs are approved yet, several are undergoing preclinical and clinical development for better selectivity and lower toxicity [159]. The field of therapeutic zinc complexes for cancer treatment is advancing swiftly, encompassing various promising classes. Notably, the thiosemicarbazone derivative COTI‐2 is in Phase 1b trials for advanced gynecologic cancers, acting as a zinc metallochaperone to regulate cellular zinc levels [160]. Zinc phthalocyanines (ZnPcs) (Figure S11) are also being developed for photodynamic therapy (PDT), utilizing light to generate ROS for targeted cancer cell death [161]. Additionally, zinc Schiff base complexes, particularly those from cysteine, showcase high potency and low toxicity [162]. Furthermore, zinc‐based NPs (ZnO NPs) are being investigated for their favorable ADMET properties and ability to specifically target cancer cells within the tumor microenvironment [163, 164]. Overall, these distinct classes demonstrate unique mechanisms and favorable characteristics, indicating a promising future in therapeutic applications.

### 7.1. Absorption and Distribution of Zinc Complexes

The cellular uptake of therapeutic zinc coordination complexes is a sophisticated process that moves well beyond simple passive diffusion. It relies on various biological and physiochemical strategies to achieve targeted delivery and overcome drug resistance [158].

#### 7.1.1. Absorption of Zinc (Zn) Complexes

The biological absorption of zinc complexes is primarily driven by active transport, carrier interactions, and endogenous metal homeostasis pathways. A primary mechanism for zinc complexes entering cells is their interaction with carrier proteins in the bloodstream, like transferrin and human serum albumin (HSA). This strategy minimizes toxicity to healthy tissues while effectively targeting cancer cells, which have a higher demand for zinc [165]. A key factor affecting zinc uptake in cancer is the dysregulation of zinc transporters (ZIP and ZnT families) in malignant tissues. These transporters often show abnormal expression levels in cancer [166, 167]. For example, ZIP10 overexpression in aggressive cancers such as breast cancer enhances zinc uptake and is associated with increased cell migration, creating both therapeutic opportunities and biological challenges [168].

#### 7.1.2. Physicochemical and Formulation Strategies of Zinc (Zn) Complexes

When a zinc complex cannot rely solely on endogenous transport proteins, its structural and formulation properties can be altered to force alternative absorption pathways. The success of zinc complexes as therapeutics is strongly influenced by the manipulation of their physicochemical properties and the design of the ligand [158]. Passive membrane diffusion is significantly influenced by the structural design of the coordinating ligand, which determines the complex’s lipophilicity. Highly lipophilic ligands, like dithiocarbamates, facilitate the zinc complex’s ability to passively diffuse across the lipid bilayer of the plasma membrane, enabling swift, unregulated absorption into cells and direct access to intracellular targets [169].

Advanced formulations of zinc metallochaperones, such as COTI‐2, have favorable absorption characteristics [160]. However, highly hydrophobic compounds like ZnPcs face significant absorption challenges. To address this, advanced encapsulation techniques, including loading into human serum albumin nanoparticle (HSA NPs), are necessary to prevent aggregation, ensure stable systemic absorption, and facilitate controlled intracellular release [161]. Alternative coordination designs, such as cysteine‐based zinc Schiff base complexes, enhance cellular absorption profiles by achieving high internalization rates in drug‐resistant cell lines, effectively bypassing multidrug resistance pumps [162]. Additionally, inorganic formulations like ZnO NPs employ active endocytosis, where the entire structure is ingested and directed to lysosomes for toxin release [163, 164].

#### 7.1.3. Distribution of Zinc Complexes

##### 7.1.3.1. Physiochemical Factors Influencing Distribution

The systemic distribution of zinc complexes is fundamentally influenced by their affinity for biological molecules and the distinct array of zinc transporters expressed across different tissues [167]. The organic ligands complexing with the zinc ion are paramount, as they dictate the complex’s bioavailability and tissue accumulation [167]. Furthermore, a high binding affinity to serum proteins such as BSA or HSA is often leveraged for effective drug delivery [170, 171]. These proteins act as natural carriers, optimizing the complex’s pharmacokinetics and enhancing therapeutic outcomes [170, 171].

#### 7.1.4. Targeted Delivery and Biological Barriers of Zinc (Zn) Complexes

Zinc complexes employ targeted strategies to address passive distribution challenges. NP formulations, such as ZnPcs and ZnO NPs, utilize the enhanced permeability and retention (EPR) effect, enabling selective accumulation in tumor tissues due to their leaky vasculature and inadequate lymphatic drainage [163, 164]. The accumulation pattern is further influenced by the NPs’ surface properties [163, 164]. Additionally, cysteine‐based zinc Schiff base complexes demonstrate effective in vivo distribution and promising tumor targeting in mouse models [162]. A major challenge in developing targeted agents is addressing tissue‐specific effects and toxicities that can limit their effectiveness, necessitating targeted drug delivery systems [158]. A key obstacle is the blood–brain barrier (BBB), as many zinc complexes struggle to cross it, requiring innovative delivery systems to achieve therapeutic levels for treating central nervous system malignancies like brain tumors [172]. The metallochaperone COTI‐2 exemplifies an advanced complex being studied in clinical trials for its effective targeting of cancerous cells [160].

### 7.2. Metabolism Profile of Zinc Complexes

#### 7.2.1. Metabolism and Stability of Zinc (Zn) Complexes

The metabolic behavior of zinc complexes is governed by the stability of the metal–ligand bond and the metabolism of the ligand [173]. Stable complexes ensure effective zinc delivery to cancerous tissues, while metabolically labile ligands facilitate rapid breakdown and release of cytotoxic zinc ions postcellular uptake [173, 174]. For example, stable ZnPcs act through light activation [175], whereas ZnO NPs dissolve in acidic environments to release toxic Zn^2+^ ions, causing mitochondrial dysfunction and cell death [163, 164].

#### 7.2.2. Oxidative Stress of Zinc (Zn) Complexes

Despite Zn^2+^ ion being redox‐inactive, zinc complexes are designed to leverage high oxidative stress in cancer cells [176]. Once inside, they induce increased ROS production primarily through mitochondrial dysfunction [177]. This excess ROS overwhelms the cell’s defenses, leading to oxidative damage and apoptosis [178]. For instance, cysteine‐based zinc Schiff base complexes disrupt cellular redox balance, influencing ROS and GSH levels to prompt cell death [162].

#### 7.2.3. Mechanisms of Cytotoxicity of Zinc (Zn) Complexes

Advanced zinc complexes are engineered to combat cancer cell survival mechanisms. These complexes inhibit antioxidant enzymes that cancer cells upregulate to withstand oxidative stress, consequently enhancing the impact of ROS and increasing susceptibility to cytotoxic effects [179, 180]. Additionally, some zinc complexes, like COTI‐2, promote cell death via apoptosis through both p53‐dependent and p53‐independent pathways by causing DNA damage and replication stress, while activating AMPK and inhibiting mTOR pathways [160].

### 7.3. Excretion Pathway of Zinc Complexes

The excretion pathway for a zinc complex is influenced by its physicochemical properties, including size, charge, and water solubility [181]. Smaller, water‐soluble complexes are efficiently eliminated through the kidneys into urine, while larger, more lipophilic compounds are generally removed via biliary excretion into the digestive tract [181]. The therapeutic zinc compounds and their formulations generally show favorable safety and excretion profiles in preclinical studies. Zinc metallochaperone (COTI‐2) demonstrates good safety and metabolism, although human data are still emerging; thus, incomplete information is available [160]. A cysteine‐based zinc Schiff base complex has favorable excretion and no observed systemic toxicity in preliminary mouse studies [161]. Biodegradable ZnO NPs can be cleared from the body [163, 164], while ZnPcs are eliminated through different pathways based on their design and size [162–164].

### 7.4. Toxicity Profile of Zinc Complexes

Therapeutic zinc complexes, such as 2Meobpy‐Zn and COTI‐2, demonstrate lower toxicity and higher selectivity against cancer cells, offering effective alternatives to conventional drugs like cisplatin [159]. For instance, 2Meobpy‐Zn is more effective against MCF‐7 breast cancer cells compared to normal NIH/3T3 cells at a concentration of 5 μM [159]. COTI‐2 has shown promising safety in Phase 1b trials with fewer side effects [160]. Additionally, cysteine‐based zinc Schiff base complexes exhibit nontoxic effects on healthy cells, highlighting their selective action [161]. Zinc ions also provide protective benefits by displacing toxic metals, reducing harmful free radicals [182].

Zinc complexes exhibit lower toxicity than platinum‐based chemotherapy but can inhibit lymphocyte function, leading to immunosuppression [183]. ZnO NPs selectively kill cancer cells by releasing toxic Zn2+ ions in lysosomes, demonstrating a favorable toxicity profile [162]. The effects of zinc supplements in cancer vary by cancer type and gender, with ligands possibly disrupting cellular functions and showing toxic properties [183, 184]. Phthalocyanines (ZnPcs) have low systemic toxicity without light, but their cytotoxicity increases in illuminated areas, especially when combined with targeting ligands [163, 164].

## 8. Comparative ADMET Characteristics and Key Insights

A comparative analysis of platinum (Pt), palladium (Pd), gold (Au), ruthenium (Ru), copper (Cu), and zinc (Zn) complexes demonstrates highly diverse ADMET profiles, as summarized in Table S3. The variation strongly highlights the justification for creating nonplatinum substitutes to overcome the severe systemic toxicity and cross‐resistance long associated with classical platinum therapy.

### 8.1. Comparison of Absorption and Distribution

Many metallodrugs experience poor oral bioavailability due to their physicochemical properties [2], necessitating intravenous administration for traditional platinum drugs like cisplatin [17]. To enhance patient convenience, orally active prodrugs such as satraplatin auranofin and modern zinc (e.g., COTI‐2 and C‐ZnSB) have been developed [62]. On the one hand, copper absorption mechanisms differ significantly based on coordination chemistry where lipophilic complexes like elesclomol utilize passive diffusion, often outperforming platinum drugs in cellular uptake, while hydrophilic variants rely on endocytosis [133–135]. Similarly, zinc oxide NPs optimize tumor internalization through endocytosis [163, 164].

All six metal complexes have unique protein‐binding interactions that affect their distribution and therapeutic effectiveness. Platinum complexes often irreversibly bind to plasma proteins like albumin, leading to inactivation and off‐target damage [10]. In contrast, palladium complexes utilize strong chelate ligands to reduce nonspecific binding and slow ligand exchange [46]. Ruthenium complexes preferentially associate with serum albumin and transferrin, mimicking iron transport [117], while gold complexes show moderate, reversible binding to serum proteins [62]. Copper depends on albumin for transport and uses ionophores for targeted delivery [131], whereas zinc complexes achieve favorable distribution through specific tumor‐targeting ligands that enhance survival and decrease toxicity [167]. Although this protein binding is essential for the distribution and transportation of drugs, it can also result in toxicity and drug inactivation, indicating a complicated interaction [10].

Cellular distribution for these metals varies significantly (Table S3). Platinum primarily uses passive diffusion and active transporters, leading to wide dispersion and off‐target damage [185]. In contrast, ruthenium complexes target transferrin receptors, taking advantage of cancer cells’ iron needs [117]. Gold and palladium complexes use nanocarrier systems to leverage the EPR effect for tumor targeting [62], while zinc complexes also benefit from EPR, ensuring effective targeting and extended therapeutic effects [163, 164].

A fundamental biological dichotomy exists between essential metals (copper and zinc), which are processed through regulated homeostatic systems to prevent deficiency or toxicity [19], and noble metals (platinum, palladium, gold, and ruthenium), treated as foreign substances [186]. Essential metals allow for more tumor‐specific synthetic complexes, while noble metals face a higher risk of off‐target accumulation and chronic toxicity due to the absence of specialized clearance pathways.

### 8.2. Comparative Metabolic Pathways and Activation Mechanisms

Each of the six metal groups exhibits a distinct primary molecular target and metabolic cascade within the intracellular environment. The design of inert prodrugs marks a significant advancement in metallodrug development, enabling selective activation in specific tumor microenvironments, such as hypoxia and low pH [113]. Ruthenium(III) and platinum(IV) complexes exemplify this by remaining inactive until reduced to their cytotoxic Ru(II) and Pt(II) forms in hypoxic tumor cells [18]. Gold(I) complexes, including auranofin, also rapidly release active components through intracellular ligand exchange [54, 187]. The unique conditions can selectively activate metal complexes within tumors, improving therapeutic effects and reducing collateral damage to healthy cells.

Among essential metals, copper complexes induce oxidative collapse via redox cycling and ligand exchange [122, 188], while zinc complexes reactivate apoptotic pathways or releasing zinc ions with specific biochemical triggers [188, 189]. This paradigm shift toward context‐dependent, multitargeted activation strategy offers promising strategy for overcoming cisplatin resistance. Future metallodrugs could be smart drugs responding to cancer cell biochemical signatures.

### 8.3. Differences in Excretion Profiles and Toxicities

The organ‐specific toxicities of chemotherapeutics are linked to their excretion pathways, and this distinguishes metal classes. Platinum compounds are largely eliminated through the kidneys and often cause nephrotoxicity and other side effects [35, 190]. Palladium has lower tissue accumulation, resulting in reduced systemic toxicity and improved cancer cell selectivity when structurally stable [46, 51]. Gold complexes are mainly eliminated via feces, minimizing nephrotoxicity and yielding higher LD_50_ values than cisplatin, although some can be toxic to the liver and spleen [62, 191]. Ruthenium compounds show varied excretion profiles with generally low toxicity, benefiting from targeted antimetastatic effects [117, 120]. Copper is primarily cleared through the hepatic‐biliary pathway, with risks of oxidative stress leading to hepatotoxicity or nephrotoxicity upon accumulation [48]. Conversely, zinc complexes utilize the body’s homeostatic controls, ensuring a favorable safety profile with minimal side effects and negligible toxicity to healthy cells [48]. Mapping diverse ADMET profiles enables the design of metallodrugs that minimize reliance on renal clearance. By choosing metals that utilize nonrenal excretion pathways, future therapeutics can then be designed to enhance tumor destruction while protecting vital organs from chronic toxicity.

## 9. Challenges in ADMET Assessment of Metallodrugs

The evaluation of metallodrugs’ ADMET properties is complex due to factors like complicated chemistry, varying metal speciation, oxidation states, and binding ligands, alongside challenges in identifying non‐DNA intracellular targets [2]. To address these issues, analytical techniques for metal distribution, speciation studies, in vitro screening, and omics approaches are utilized to elucidate mechanisms of action [192]. Furthermore, new computational techniques and machine learning models are being integrated to enhance predictions of ADMET features and streamline the development of metal‐based drugs [193].

### 9.1. Challenges to Analytical Techniques

Metallodrugs encounter major challenges in the human body due to interactions with endogenous molecules such as chloride, phosphate, carbonate anions, and serum proteins such as human serum albumin (HSA) and apo‐transferrin [35, 194]. These interactions can chemically alter metallodrugs, impacting their and composition biological function [35]. The complex nature of biological matrices complicates the design and development of metallodrugs for therapeutic applications [195].

The analytical process of speciation analysis involves determining and quantifying distinct chemical species of an element in a sample. In biological systems, it is vital for differentiating metal complexes, free metal ions, and colloidal species [196]. Variations in speciation complicate metallodrug analysis due to the instability of chemical forms affecting biological activity, as well as frequent changes from ligand exchange, redox reactions, and interactions with biological matrices [197]. Effective analytical techniques must isolate and measure these dynamic species without alteration, although analyzing such biological systems remains a significant challenge.

Accurate in vivo speciation analysis is challenging due to biological sample complexity, low metal‐adduct concentrations, and the need to preserve species identity [198, 199]. This complexity complicates the application of in vitro findings to in vivo conditions, as variations in reaction media and analytical conditions arise [200]. Indirect methods are essential to avoid species degradation, as direct measurements in living organisms are often unfeasible [201]. The instability of certain metallodrugs and their adducts during sample preparation can alter or dissociate drug species, risking data integrity in ADMET assessment [200].

Translating drug therapeutic qualities into clinical practice is hindered by uncertainties in determining specific chemical forms or metabolites. Advances in analytical chemistry are essential for metallodrug development, necessitating investment in novel, sensitive, and species‐preserving analytical techniques for breakthroughs.

### 9.2. Challenges of Redox Activity on ADMET Profiles

The capacity of transition metal ions, such as platinum, palladium, gold, and ruthenium, to alternate between various oxidation states significantly influences their cellular interactions and ADMET profiles [9]. Metallodrugs, which act as prodrugs, rely on redox processes, particularly in cancer cells, where reducing environments can convert Pt(IV) and Ru(III) to their more active forms, Pt(II) and Ru(II). This strategy aims to activate the drugs selectively at tumor sites; however, disruption on cellular redox homeostasis generates ROS, which can cause cytotoxicity against off‐target toxicity to healthy tissues [113]. Redox processes similarly can significantly impact a drug’s lipophilicity, binding to proteins, and subsequent cellular uptake [113]. Moreover, the detoxification process that affects the body’s excretion of metallodrugs is redox‐related conjugation events, especially with endogenous thiols like GSH [104].

The broad pharmacokinetic and pharmacodynamic behavior of metallodrugs is determined by the basic, overarching concept of redox chemistry. The side effect of drugs is intricately linked to their mechanisms of action, influencing their activation, cellular targets, therapeutic efficacy, and toxicity profiles. Understanding and controlling redox transformations is crucial for rational drug design, as metallodrug design differs from organic design, requiring deep inorganic chemistry principles.

### 9.3. Challenges in Clinical Translation

Preclinical metallodrugs face challenges such as severe toxicity, drug resistance, poor bioavailability, and lack of selectivity, leading to limited clinical success and higher doses for in vivo models [2, 202, 203]. A complex interaction of ADMET difficulties is responsible for this translational gap, which is often referred to as the “valley of death” in drug development. Key challenges include but not limited to lack of clear distinction between therapeutic and toxic doses causing significant toxicity, resistance, and off‐target effects [204]; instability in systemic circulation leading to premature activation or degradation of prodrugs [27]; poor water solubility and low bioavailability; improper in vivo distribution and rapid elimination [205]; failure to obey Lipinski’s rule of five [2]; and unpredicted systemic toxicity [206].

Many metallodrugs show great promise in vitro but fail to translate in vivo or clinically due to complex ADMET challenges. The problems are essentially related to drug safety and disposition in a live system, not only efficacy. This translation is made even more difficult by the shortcomings of conventional predictive models for metallodrugs. A comprehensive, the predictive ADMET assessment framework is needed for metallodrugs, incorporating advanced experimental and computational tools from the initial stages of drug discovery.

## 10. Emerging Trends and Future Research Directions

A deeper understanding of ADMET principles has facilitated the advancement of metal‐based anticancer drugs by addressing issues like poor targeting and side effects [3]. The integration of these principles with advanced technologies such as nanodelivery systems, computational modeling, and artificial intelligence is leading to the development of more effective and personalized therapies [207]. Future research targets to enhance therapeutic potential while overcoming current limitations using these modern approaches.

### 10.1. Novel Delivery Systems and Targeted Approaches

Precision medicine in metallodrug development focuses on sophisticated delivery systems to enhance ADMET profiles, reduce damage to healthy tissues, and mitigate toxicities common in first‐generation drugs. Advanced polymeric NPs, including AP5280, ProLindac, and NC‐6004, are being developed to improve platinum accumulation in tumors and decrease systemic side effects [12]. Additionally, gold NPs and cyclodextrin inclusion complexes are being investigated to enhance solubility and bioavailability in copper complexes [27, 208].

Ligand functionalization involves attaching metallic compounds to biological carriers to enhance specificity, improve direct drug delivery to cancer cells, and address ADMET challenges [17]. The focus of such research is on developing nontoxic, selectively activated prodrugs that can be triggered by tumor‐expressed enzymes or external stimuli, which aim to increase selectivity and lessen systemic toxicity [11]. The future of metallodrugs should emphasize rational design, combining the drug with precise delivery mechanisms to maximize therapeutic effects while minimizing damage to healthy tissues.

### 10.2. Advanced Analytical and Computational Methods

ADMET analysis for metallodrugs, enhanced by AI‐driven predictive modeling, is transforming drug discovery by addressing pharmacokinetics and toxicity issues such as clinical drug failures [209, 210]. Key experimental domains such as metalloproteomics, genomics, and molecular biology are essential for understanding the properties of effective antitumor agents [211–213]. The most effective strategy combines experimental and computational approaches such as electron paramagnetic resonance and mass spectrometry together theoretical techniques such as density functional theory (DFT) and protein–ligand docking [214–216]. This integrated approach should also include high‐throughput omics to identify cellular metabolism disruptions caused by metallodrugs, moving past single‐target analyses [217–219]. Additionally, advancements in metal‐specific analytical techniques, including synchrotron x‐ray spectroscopy and enhanced mass spectrometry, are vital for monitoring metal speciation and distribution in complex biological systems [196, 220, 221].

The future of ADMET analysis for metallodrugs is expected to rely on an interdisciplinary approach, incorporating computational models and high‐resolution analytical data to improve prediction accuracy in drug discovery. By using advanced tools, researchers can create metal complexes with optimized properties for targeted therapy, enhancing tumor accumulation and minimizing off‐target effects, ultimately maximizing therapeutic index and overcoming current limitations in metal‐based anticancer agents.

## 11. Conclusions

The comprehensive ADMET profiling of metallodrugs confirms that while platinum (Pt) complexes like cisplatin, having been widely used as an anticancer drug, established the foundational efficacy through DNA binding, their clinical utility is severely compromised by high systemic toxicity and the ubiquity of acquired tumor resistance. This necessitates a strategic shift toward exploiting nonplatinum metal complexes, namely, palladium (Pd), gold (Au), ruthenium (Ru), copper (Cu), and zinc (Zn), which uses diverse and nuanced ADMET pathways to achieve improved selectivity and lower acute toxicity.

A comparative ADMET review highlights the unique benefits of each metal class. Ruthenium complexes leverage cancer‐specific characteristics, such as hypoxia and transferrin receptors, to act as prodrugs with notable antimetastatic properties. Gold complexes show exceptional versatility by targeting non‐DNA pathways, such as TrxR and the proteasome, and their preferential fecal/biliary excretion offers a critical pathway to mitigate the renal toxicity associated with platinum. Copper and zinc complexes are particularly promising, harnessing their role in essential biological processes; copper agents often use ionophores (e.g., elesclomol) for targeted mitochondrial delivery and ROS generation, leading to unique cell death mechanisms like ferroptosis, while zinc complexes (e.g., COTI‐2) demonstrate favorable oral bioavailability and selective p53 reactivation.

Despite the significant preclinical promise of nonplatinum metallodrugs, a persistent gap remains between laboratory findings and clinical success, often stemming from unpredictable ADMET properties and complex metal speciation within biological systems. Overcoming this challenge and moving beyond the Pt scaffold demands a data‐driven, interdisciplinary strategy focused squarely on optimizing ADMET properties. Future therapeutic success hinges on the rational design of complexes that can effectively utilize passive diffusion or specific tumor‐overexpressed transporters for uptake, function as prodrugs selectively activated by the tumor microenvironment (e.g., hypoxia, ROS levels), and be rationally engineered to favor nonrenal excretion (fecal/biliary) to circumvent major organ toxicities. Ultimately, the integration of cutting‐edge analytical techniques, such as metalloproteomics and metal‐specific imaging, alongside AI‐driven predictive modeling, is crucial for developing metallodrugs with enhanced stability, controlled activation, and superior therapeutic indices that align with the goals of medicine precision.

## Funding

No funding was received for this manuscript.

## Conflicts of Interest

The authors declare no conflicts of interest.

## Supporting Information

Additional supporting information can be found online in the Supporting Information section.

## Supporting information


**Supporting Information 1** Table S1: Key proteins interacting with platinum‐based drugs and their associated consequences. Table S2: common toxicities and resistance mechanisms of platinum‐based anticancer drugs. Table S3: summary of the key ADMET properties of Pd complexes. Table S4: summary of ADMET characteristics of Pt, Pd, Au, and Ru anticancer complexes.

**Supporting Information 2** Figure S1: clinically used platinum complexes. Figure S2: Pt complexes under clinical investigation. Figure S3: structure of satraplatin. Figure S4: the types of DNA adducts formed by cisplatin. A) Intrastrand cross‐links formed by cisplatin with DNA. a) 1,2‐d(GpG) intrastrand cisplatin cross‐links. These are the majority adduct formed by the reaction of cisplatin with DNA. b) 1,2‐d(GpA) intrastrand. Figure S5: PdL and 9A Pd complexes. Figure S6: palladium complexes, Pd–Py–OEt and Pd–Py–NEt2, similar to cisplatin. Figure S7: representation of the AuNP structure with a gold core and surface functionalization. The particles feature a (1.2–1.6 nm) diameter gold core capped by an organic layer composed of a {50:50}C mixture of thiolated alpha‐galactose derivative and thiol PEG amine, attached via gold–sulfur (Au–S) bonds (Tzelepi et al., 2016). Figure S8: sample gold complexes that have shown anticancer properties. Figure S9: structures of auranofin and dual‐gold(I) complex (QB1561). Figure S10: some of the ruthenium complexes under clinical trials for antitumor activity. Figure S11: Ru complexes showing good in vitro and in vivo outcomes. Figure S12: structures of RAPTA –T and –C. Figure S13: chemical structures of disulfiram (DSF), diethyldithiocarbamate (DDC), and bis‐diethyldithiocarbamate {Cu(DDC)2}. Figure S14: structure of zinc phthalocyanine.

## Data Availability

The data that support the findings of this study are available in the supporting information of this article.
