# Supplementary material for: ADMET Profiling of the Metallodrugs: A Comparative Review of Platinum, Palladium, Gold, Ruthenium, Copper, and Zinc Anticancer Complexes
Source: Bioinorg Chem Appl. 2026 Aug 12;2026:9578818. doi: 10.1155/bca/9578818 (PMC13462909; doi:10.1155/bca/9578818)
Supplement: Supplementary file 1 [file BCA-2026-9578818-s001.docx]

**List of Figures**

[Figure S1: Clinically used platinum complexes	2](#_Toc225015740)

[Figure S2: Pt-Complexes under clinical investigation	2](#_Toc225015741)

[Figure S3: Structure of Satraplatin	2](#_Toc225015742)

[Figure S4 The types of DNA adducts formed by cisplatin. A) Intrastrand cross-links formed by cisplatin with DNA. a) 1,2-d(GpG) intrastrand cisplatin cross-links. These are the majority adduct formed by the reaction of cisplatin with DNA. b) 1,2-d(GpA) intrastrand	3](#_Toc225015743)

[Figure S5: PdL and 9A Pd - Complexes	3](#_Toc225015744)

[Figure S6: Palladium complexes, Pd-Py-OEt and Pd-Py-NEt_2_, similar to cisplatin	4](#_Toc225015745)

[Figure S7: Representation of the AuNP Structure with a Gold Core and Surface Functionalization. The particles feature a (1.2-1.6 nm) diameter gold core capped by an organic layer composed of a {50:50}C mixture of **thiolated alpha-Galactose derivative** and **thiol PEG amine**, attached via gold-sulfur (Au-S) bonds (Tzelepi et al., 2016).	5](#_Toc225015746)

Figure S8: sample gold complexes that have shown anticancer properties………………………………5

[Figure S9: Structures of Auranofin and dual-gold(I) complex (QB1561)	6](#_Toc225015747)

[Figure S10: Some of the ruthenium complexes under clinical trials anti-tumor activity	7](#_Toc225015748)

[Figure S11: Ru complexes showing good *in vitro* and *in vivo* outcomes	8](#_Toc225015749)

[Figure S12: Structures of RAPTA –T and –C	9](#_Toc225015750)

[Figure S13: Chemical Structures of Disuliram (DSF), Diethyldithiocarbamate (DDC), and Bis-diethyldithiocarbamate {Cu(DDC)_2_}	9](#_Toc225015751)

[Figure S14: Structure of Zinc phthalocyanine	10](#_Toc225015752)

Figure S1: Clinically used platinum complexes

Figure S2: Pt-Complexes under clinical investigation

Figure S3: Structure of Satraplatin

Figure S4 The types of DNA adducts formed by cisplatin. A) Intrastrand cross-links formed by cisplatin with DNA. a) 1,2-d(GpG) intrastrand cisplatin cross-links. These are the majority adduct formed by the reaction of cisplatin with DNA. b) 1,2-d(GpA) intrastrand

PdL

Figure S5 PdL and 9A Pd - Complexes

Figure S6: Palladium complexes, Pd-Py-OEt and Pd-Py-NEt_2_, similar to cisplatin

Figure S7: Representation of the AuNP Structure with a Gold Core and Surface Functionalization. The particles feature a (1.2-1.6 nm) diameter gold core capped by an organic layer composed of a {50:50}C mixture of **thiolated alpha-Galactose derivative** and **thiol PEG amine**, attached via gold-sulfur (Au-S) bonds (Tzelepi et al., 2016).

Gold(III)dithiocarbamate

Figure S8: sample gold complexes that have shown anticancer properties

Figure S9: Structures of Auranofin and dual-gold(I) complex (QB1561)

Figure S10: Some of the ruthenium complexes under clinical trials anti-tumor activity

[Ru (dip)_2_(PPβC)]PF_6_

Figure S11: Ru complexes showing good *in vitro* and *in vivo* outcomes

Figure S12: Structures of RAPTA –T and –C

Figure S13: Chemical Structures of Disuliram (DSF), Diethyldithiocarbamate (DDC), and Bis-diethyldithiocarbamate {Cu(DDC)_2_}

Figure S14: Structure of Zinc phthalocyanine
