# Supplementary material for: ADMET Profiling of the Metallodrugs: A Comparative Review of Platinum, Palladium, Gold, Ruthenium, Copper, and Zinc Anticancer Complexes
Source: Bioinorg Chem Appl. 2026 Aug 12;2026:9578818. doi: 10.1155/bca/9578818 (PMC13462909; doi:10.1155/bca/9578818)
Supplement: Supplementary file 2 [file BCA-2026-9578818-s002.docx]

**Supplementary materials**

**List of tables**

[Table S1: Key Proteins Interacting with Platinum-Based Drugs and Their Associated Consequences	1](#_Toc225016094)

[Table S2: **Common Toxicities and Resistance Mechanisms of Platinum-Based Anticancer Drugs**	1](#_Toc225016095)

[Table S3: Summary of the key ADMET properties of Pd-complexes	2](#_Toc225016096)

[Table S4: Summary of ADMET Characteristics of Pt, Pd, Au, and Ru Anticancer Complexes	3](#_Toc225016097)

Table S1: Key Proteins Interacting with Platinum-Based Drugs and Their Associated Consequences

| Protein code | Description | Role | Consequences of interacting with Pt-drugs | References |
| --- | --- | --- | --- | --- |
| HMGB1 | Non-histone chromosome-binding protein | Stimulate immune cells and trigger inflammatory response | bind to platinum–DNA adducts, initiating inflammatory responses | (Yuan et al., 2024);  (Oberoi et al., 2013) |
| P53 | Tumor suppressor protein | Cell cycle arrest, cell senescence, DNA repair, metabolic adaptation and cell death | Platinum drugs can activate p53-mediated pathways, causing death of normal cells | (Aubrey et al., 2018);  (Oberoi et al., 2013) |
| Parp-1 | Poly(ADP-ribose) polymerase | Recruits DNA repair proteins to the site of DNA that needs repair | Platinum drugs can indirectly interact with PARP-1, initiating unnecessary DNA repair pathways | (Spiegel et al., 2021);  (Oberoi et al., 2013) |
| YB-1 | Nuclear expression of Y box-binding protein | Modulate drug resistance mechanisms | Associated with Pt-drug resistance | (Yahata et al., 2002)  (Oberoi et al., 2013) |
| TBP and hMSH2 | Transcription initiation factor and mismatch repair protein | Recognize platinum-induced DNA lesions | their interactions may also affect off-target pathways related to cellular stress and toxicity | (Oberoi et al., 2013) |

Table S2: Common Toxicities and Resistance Mechanisms of Platinum-Based Anticancer Drugs

| **Complex** | **Primary toxicities** | **Key resistance mechansim** | **Unique ADMET** |
| --- | --- | --- | --- |
| Cisplatin | Nephrotoxicity, Neurotoxicity, Ototoxicity, Myelosuppression, Emetogenesis  (Gold & Raja, 2025; Hartmann & Lipp, 2003) | Reduced accumulation, elevated Glutathione, increased DNA repair (MMR deficiency), overexpression of anti-apoptotic protiens (Tchounwou et al., 2021) | DNA-binding via aquation; Extensive, irreversible protein binding (Qin et al., 2019) |
| Carboplatin | Myelosuppression, hematotoxicity  (Cheng et al., 2017) | Cross-resistance with cisplatin  (Rabik & Dolan, 2007; Tchounwou et al., 2021) | Slower aquation rate; reduced nephrotoxicity; lower, more reversible protein binding (Qin et al., 2019) |
| Oxaliplatin | Peripheral Neurotoxicity  (Saif & Reardon, 2005) | Avoids cisplatin resistance by overcoming mitochondrial cell death defects (Gourdier et al., 2002) | Slower aquation rate; high protein binding; binds to erythrocytes (Qin et al., 2019) |
| Satraplatin | Myelosuppression, Gastrointestinal (GI) disorders  (Bhargava & Vaishampayan, 2009) | Decreased drug absorption, increased clearance/breakdown, and tolerance to DNA damage (Deng, 2023) | Oral prodrug (Pt (IV) to Pt (II) reduction); No nephrotoxicity/neurotoxicity/ototoxicity; No hydration required  (Qin et al., 2019) |
| heptaplatin | nephrotoxicity, myelosuppression (bone marrow damage), and stomach upset. (Liu et al., 2014) | Multifaceted drug resistance mechanisms and cell survival strategies  (Choi et al., 2004) | Rapid bonding with DNA and cellular detox agents (GSH) (Guan et al., 2019) |
| Miriplatin | Low kidney toxicity profile (Imai et al., 2011) | Resistance to cell death and platinum-based drugs (Hanada et al., 2010) | Cross-linking by a nucleophilic-binding DNA sites (Imai et al., 2011) |

Table S3: Summary of the key ADMET properties of Pd-complexes

| ADMET Property | Key Findings | Implication/Challenge | References |
| --- | --- | --- | --- |
| Absorption & Distribution (A&D) | Influenced by lipophilicity (Log P_o/w_) and specific formulation (e.g., self-assembling nanostructures for tumors). | Favorable for oral absorption (acceptable Log P values). Some forms show better selectivity (e.g., Pd2-Spm for TNBC cells) and lower overall tissue accumulation than Pt(II) equivalents. | Mbugua et al., 2020 |
| Stability & Kinetics (A&D/M) | Excessive lability (instability) is a major challenge. Pd complexes have 10^5^ times faster ligand exchange kinetics than Pt complexes. | Requires specific chelating ligands to improve stability and slow hydrolysis rates. | (Jahromi et al., 2016; Kuijpers & Blom, 2021; Onunga et al., 2020) |
| Metabolism (M) | Causes profound metabolic disruption and cellular stress. | Induces apoptosis. Depletes the antioxidant glutathione and inhibits key antioxidant enzymes. Leads to oxidative stress and ER stress | (Carneiro et al., 2020; Wang & Youle, 2009) |
| Excretion (E) | Primarily involves biliary and renal pathways, but mechanisms are not well understood. | Persistence in the body Effects of long-term accumulation and chronic toxicity not known | (Vojtek et al., 2021) |
| Toxicity & Selectivity (T) | 10x less toxic than platinum compounds. Shows increased cytotoxic activity and selectivity for various cancer cell lines. | Selectivity is improved by using strong, tightly bound chelate ligands | (Jahromi et al., 2016; Lima et al., 2025; Scattolin et al., 2021) |

Table S4: Summary of ADMET Characteristics of Pt, Pd, Au, and Ru Anticancer Complexes

| Metal | Examples | Absorption | Distribution | Metabolism | Excretion | Toxicity |
| --- | --- | --- | --- | --- | --- | --- |
| **Platinum (Pt)** | Cisplatin, Carboplatin, Oxaliplatin, Satraplatin | (Cisplatin, Carboplatin, Oxaliplatin) less bioavailable; Oral prodrug (Satraplatin) and Nanoparticle delivery explored | High, irreversible protein binding (Cisplatin, Oxaliplatin); Lower, reversible protein binding (Carboplatin); CTR1/OCT-mediated uptake; Accumulates in liver, kidneys, tumors | Aquation to active species; DNA binding (major target); Prodrug (Pt (IV) to Pt (II)) activation; Glutathione conjugation for detoxification | Renal | Nephrotoxicity, Neurotoxicity, Myelosuppression, Ototoxicity, Emetogenesis |
| **Palladium (Pd)** | Pd_2_Spm, Pd-Py-OEt | Acceptable Log P for bioavailability; Nanoparticle delivery explored | Rapid ligand exchange (challenge for intact delivery); Lower overall tissue accumulation than Pt; Selective for some cancer cells (e.g., TNBC); Tightly bound chelate ligands reduce non-specific binding | Less clear primary targets; Metabolic effects on cell death, antioxidant defense; Catalytic activity (e.g., amide hydrolysis) | Less detailed, likely renal/biliary | Generally reduced toxicity (10x less than Pt); Enhanced selectivity for cancer cells |
| **Gold (Au)** | Auranofin, Au(III) dithiocarbamates, AuNPs | Oral prodrug (Auranofin); Balanced lipophilicity/hydrophilicity; Nanoparticle delivery (size-tunable, targeted) | Moderate BSA binding; Protein-bound in serum; PEG-AuNPs accumulate in liver/spleen; Targeted AuNPs via antibodies | Prodrug activation; TrxR inhibition (Auranofin); Proteasome targeting (Au(III) dithiocarbamates); Mitochondrial dysfunction; Some act as antioxidants | Primarily fecal (>89%) for some complexes; Minimal renal excretion | Auranofin toxicity issues; Some AuNPs show late toxic effects; Generally good selectivity, lower organ/acute toxicity than Pt |
| **Ruthenium (Ru)** | NAMI-A, KP1019, RAPTA | Oral (some prodrugs); Nanoparticle delivery | Preferential uptake via transferrin receptors; Strong association with serum albumin/transferrin; Variable cellular internalization (NAMI-A extracellular, KP1019 intracellular); Small, low clearance, long half-life (KP1019) | Prodrug (Ru(III) to Ru(II) activation by reduction in hypoxic tumors; Ligand exchange kinetics similar to Pt; Diverse mechanisms (DNA binding, ROS generation, ER stress); NAMI-A antimetastatic focus | Variable; Low renal excretion (KP1019); Rapid renal clearance (NAMI-A)  RAPTA: renal as wel biliary | Low systemic toxicity; Reduced cytotoxicity vs. cisplatin; Some unspecific delivery toxicity; Antimetastatic properties (NAMI-A) |
| **Copper Cu** | Casiopeínas, Elesclomol, Disulfiram/Cu  Thiosemicarbazone Copper Complexes (Cu-ATSM)  HydroCuP®, (phosphino copper(I) complex) | lipophilic complexes use (Elesclomol, Disulfiram/Cu) passive diffusion (often surpassing Pt drugs). Hydrophilic complexes may use endocytic pathways. | Passive penetration into cytoplasm via lipophilicity; Active transport via ionophores for intracellular targeting (Elesclomol/Cu). Binds to plasma proteins (albumin) for systemic transport (Disulfiram/Cu) | Activation via redox chemistry Redox-cycling [Cu(II)/Cu(I)], ROS production  Intracellular transformation: Undergo ligand exchange (Disulfiram/Cu, Casiopeínas); Induce oxidative stress, ferroptosis (Elesclomol), and inhibit Top I/II (TSC complexes) | Primarily Biliary/Fecal  Hepatic Pathway: Complexes/metabolites are released into bile for elimination in feces; this is regulated by ATP7B. Renal Excretion is minor, typically for small, hydrophilic fragments in urine. Complex design (size, lipophilicity) | oxidative stress in healthy cells. (hepatotoxicity, nephrotoxicity from accumulation), Genotoxicity (ROS-induced DNA damage), Hematotoxicity, and off-target effects |
| **Zinc (Zn)** | COTI-2 (Thiosemicarbazone)  Zinc Phthalocyanines (ZnPcs) for PDT  Cysteine-based Zn Schiff Base (C-ZnSB)  Zinc Oxide Nanoparticles (ZnO NPs) | Oral bioavailability, cellular uptake (COT-2; C-ZnSB)\  Nanoparticle delivery, enhanced uptake (ZnPcs);  Endocytosis uptake, tumor microenvironment (ZnONPs) | Tumor targeting (COT-2);  EPR effect, tumor accumulation (ZnPcs, ZnONPs);  Effective tumor targeting, prolonged survival (C-ZnSB) | p53 reactivation, apoptosis induction. (COT-2;  Light-activated ROS, oxidative stress (ZnPcs);  Redox disruption, apoptosis induction (C-ZnSB);  Zinc ion release, oxidative stress (ZnONPs) | Favorable clearance, low side effects. (COT-2);  Nanoparticle clearance (ZnPcs);  Favorable safety profile (C-ZnSB);  Biodegradable clearance, favorable safety (ZnONPs) | Selective cytotoxicity, well-tolerated (COT-2);  Localized phototoxicity, low systemic risk (ZnPcs)  Selective cytotoxicity, non-toxic to normal cells (C-ZnSB);  Selective toxicity, tumor specificity (ZnONPs) |
